# Osteoblast Lineage Cells Play an Essential Role in Periodontal Bone Loss Through Activation of Nuclear Factor-Kappa B

**DOI:** 10.1038/srep16694

**Published:** 2015-12-15

**Authors:** Sandra Pacios, Wenmei Xiao, Marcelo Mattos, Jason Lim, Rohinton S. Tarapore, Sarah Alsadun, Bo Yu, Cun-Yu Wang, Dana T. Graves

**Affiliations:** 1 Department of Periodontics, School of Dental Medicine, University of Pennsylvania, Philadelphia, PA; 2 Department of Periodontology, School and Hospital of Stomatology, Peking University, Beijing, China; 3 Division of Oral Biology and Medicine, School of Dentistry, University of California, Los Angeles, CA

## Abstract

Bacterial pathogens stimulate periodontitis, the most common osteolytic disease in humans and the most common cause of tooth loss in adults. Previous studies identified leukocytes and their products as key factors in this process. We demonstrate for the first time that osteoblast lineage cells play a critical role in periodontal disease. Oral infection stimulated nuclear localization of NF-κB in osteoblasts and osteocytes in the periodontium of wild type but not transgenic mice that expressed a lineage specific dominant negative mutant of IKK (IKK-DN) in osteoblast lineage cells. Wild-type mice were also susceptible to bacteria induced periodontal bone loss but transgenic mice were not. The lack of bone loss in the experimental group was linked to reduced RANKL expression by osteoblast lineage cells that led to diminished osteoclast mediated bone resorption and greater coupled new bone formation. The results demonstrate that osteoblast lineage cells are key contributors to periodontal bone loss through an NF-κB mediated mechanism.

Periodontal disease affects the tissues that surround and support the tooth^1^,^2^. It is the most common osteolytic disease in humans and the most common cause of tooth loss in adults^3^. Periodontitis is initiated by a biofilm that forms on the tooth surface and induces an inflammatory response in connective tissue leading to the stimulation of osteoclasts and periodontal bone loss^4^,^5^. Periodontal infection stimulates the innate and adaptive immune response and the production of cytokines such as tumor necrosis factor and ligand for receptor activator of NF-κB (RANKL) that induce osteoclastogenesis^1^,^4^,^6^,^7^,^8^,^9^. We have postulated that the impact of inflammation on osteoblast lineage cells is an essential aspect of periodontitis^1^ but as of yet there is no proof of this concept.

Osteoblast lineage cells consist of osteoblasts and osteocytes. Osteoblasts produce bone matrix proteins to form osteoid and may become trapped during bone formation to further differentiate to osteocytes or undergo apoptosis^10^. Osteocytes constitute the most abundant bone cell population and are important regulators of bone remodeling, influencing both osteoblast and osteoclast function^10^,^11^. Inflammation affects osteoblast lineage cells through the transcription factor nuclear factor-kappa B (NF-κB)^12^. There are two general pathways of NF-κB activation, canonical and alternative. Many different stimuli, including inflammatory cytokines and toll-like receptors activate the canonical NF-κB pathway. The alternative pathway is activated in response to a small subset of TNF family members. NF-κB is important in bone formation. Induction of osteoporosis by ovariectomy stimulates osteoporosis that is significantly reduced in transgenic mice that express a dominant negative mutant of IKK, which inhibits NF-κB in osteoblast lineage cells^13^. These mice have greater trabecular bone mass compared to controls due to increased osteoblast activity^13^.

To investigate the role of NF-κB in osteoblast lineage cells in periodontal disease we examined mice with a dominant negative inhibitor of NF-κB under the control of a 2.3 kb regulatory unit of the collagen 1α1 promoter^13^. This promoter element restricts expression to osteoblasts and osteocytes^14^,^15^. Periodontitis was induced by oral inoculation of periodontal pathogens in a murine model that recapitulates the critical events of human periodontitis^16^. Surprisingly we found that bacteria-induced periodontal bone loss was completely blocked in in transgenic mice with inhibition of NF-κB in osteoblast lineage cells measured by microCT and histologically. We demonstrate that osteoclast formation is significantly reduced and bone formation enhanced in experimental mice demonstrating the importance of this cell lineage in the initiation and progression of periodontal bone loss. These data are the first to demonstrate that osteoblast lineage cells play an essential role in periodontal disease and indicate that they may be important therapeutic targets in the prevention and treatment of periodontitis. Moreover, they provide new insight into inflammation-induced bone loss, which is less well understood than physiological bone resorption^17^.

## Results

### Inhibiting NF-κB activation prevents bacteria-induced periodontal bone loss

**Figure 1:**
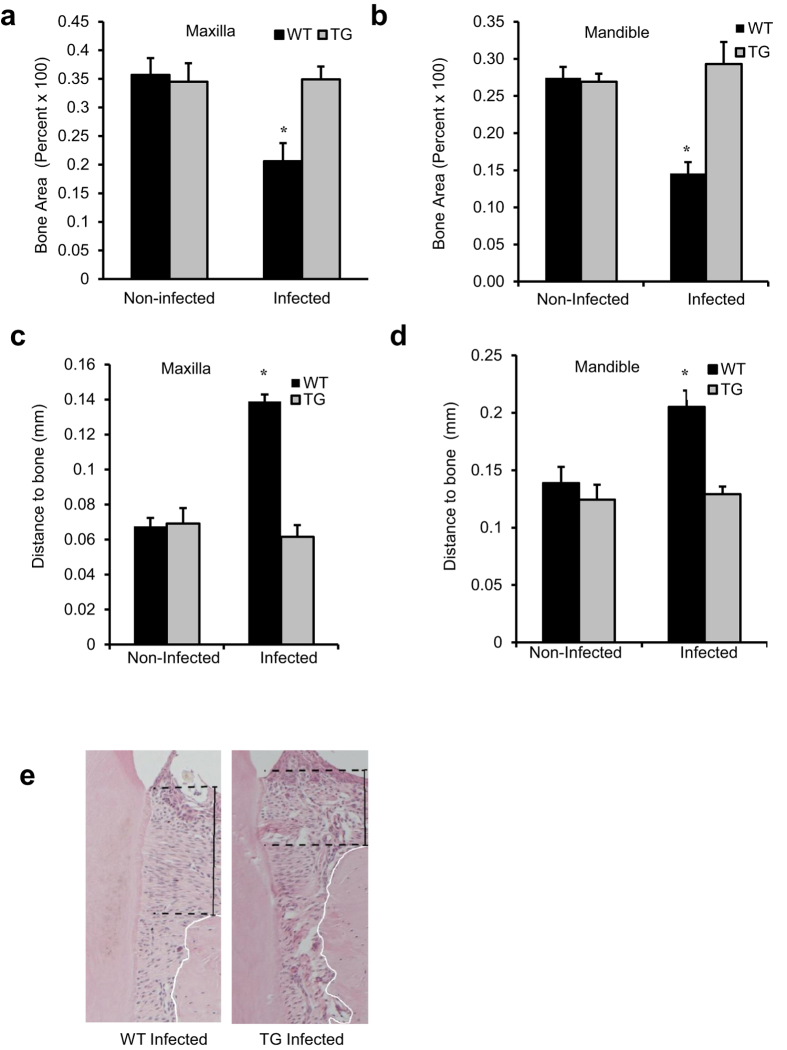
Inhibiting NF-κB activation in osteoblast lineage cells block periodontal bone loss induced by inoculation of periodontal pathogens. Periodontal disease was initiated in IKK-DN transgenic mice (TG) or wild-type (WT) control mice by oral inoculation of the periodontal pathogens *P. gingivalis* plus *F. nucleatum* or vehicle alone. Mice were euthanized 6 weeks after oral inoculation. (**a**,**b**) MicroCT analysis of bone area between the molars in the mandible and maxilla. (**c**–**e**) Distance from a reference point on the tooth surface (cemento-enamel junction) to crest of bone in hematoxylin and eosin stained sections between the molars in the mandible and maxilla. ^+^significantly different in infected compared to matched non-infected group; ^*^significantly different in infected TG compared to infected WT (P < 0.05).

MicroCT analysis demonstrates that oral infection induced a 42–45% loss in periodontal bone in both the maxilla and mandible of wild type (WT) mice (P < 0.05) (Fig. 1a,b). In contrast to normal mice, no bone loss was observed in Col1α1.IKK-DN transgenic (TG) mice. Similar results were obtained by histologic analysis. Induction of periodontal disease by bacterial inoculation caused a 2-fold loss in bone height in normal mice compared to baseline (Fig. 1c–e). However in TG mice periodontal infection caused no loss of bone height (P < 0.05).

### Periodontal infection induces NF-κB activation in osteoblasts and osteocytes but not gingival cells

**Figure 2:**
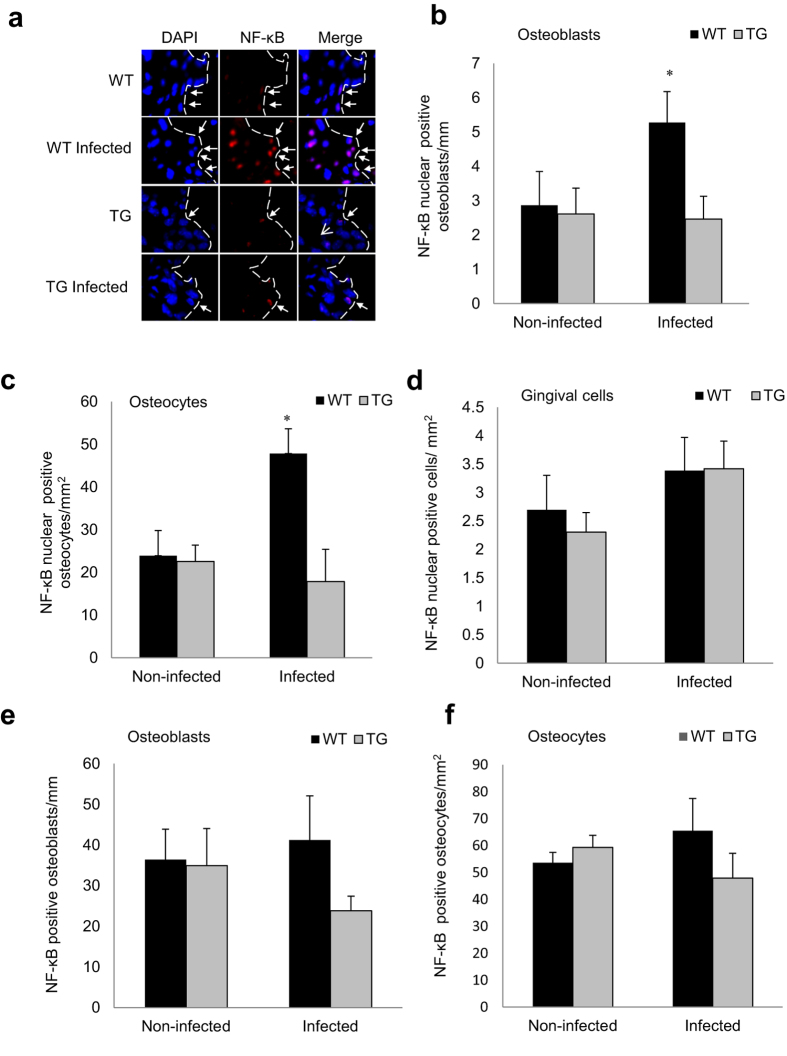
Periodontal infection stimulates NF-κB nuclear localization in osteoblasts and osteocytes, which is blocked in experimental mice. Periodontal disease was induced in wild-type and IKK-DN mice by oral inoculation of bacteria. (**a–c**) Nuclear NF-κB was measured by immunofluorescence by co-localization of NF-κB p65 immunostaining (red) and DAPI (blue) nuclear staining. Localization to bone-lining osteoblastic cells (indicated by arrows) was determined in cells with a cuboidal appearance and with a nucleus that was not fusiform or parallel to the bone surface and expressed as the number per bone length. Nuclear NF-κB was measured in osteocytes in bone matrix and expressed as the number per bone area. (**d**) Nuclear NF-κB was measured in cells located in the gingival connective tissue consisting primarily of fibroblasts and leukocytes and normalized per gingival area. (**e**,**f**) Cytoplasmic NF-κB p65 was examined by immunofluorescence by the presence of non-nuclear immunostaining in in osteoblasts and osteocytes in DAPI counterstained sections and normalized per length for osteoblasts and per area for osteocytes. Matched control antibody was negative. ^+^significantly different in infected compared to matched non-infected group (P < 0.05).

**Figure 3:**
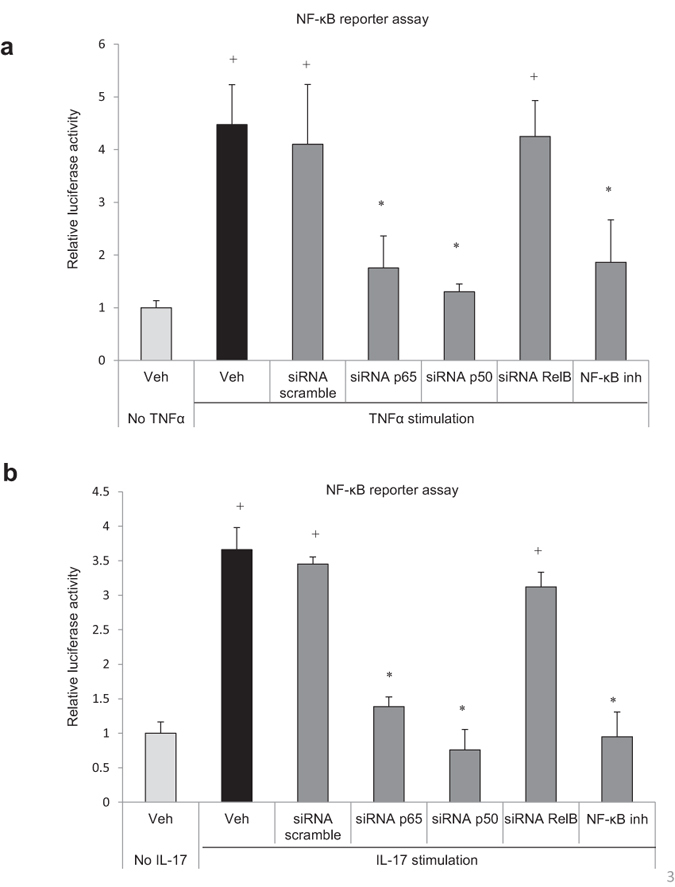
TNF and IL-17 stimulate NF-κB activity in osteoblasts. Cells were transfected with a NF-κB luciferase construct without or with transfection by scrambled siRNA or siRNA specific for NF-κB p50, p65 or relB subunits or with NF-κB inhibitor and then stimulated with (**a**) TNF or (**b**) IL-17 overnight. Luciferase activity was normalized by renilla control and expressed as relative luciferase units. ^*^significantly different compared with unstimulated cells (P < 0.05); ^+^significantly different compared stimulated control (P < 0.05).

Immunofluorescent analysis was carried out to measure NF-κB nuclear localization, which is indicative of NF-κB activation. Periodontal inoculation stimulated a 3-fold increase in NF-κB nuclear localization in osteoblasts (Fig. 2a,b, Supplemental Fig. S1 online), and 2.5-fold higher increase in osteocytes in WT compared to TG mice (P < 0.05) (Fig. 2c). In contrast NF-κB nuclear localization in gingival cells, consisting predominantly of fibroblasts and leukocytes, was stimulated by infection in both wild-type and transgenic mice (Fig. 2d). Total positive NF-κB osteoblasts (Fig. 2e) and osteocytes (Fig. 2f) were also counted. As NF-κB is widely expressed and retained in the cytoplasm the number of positive was high in all groups with or without infection. TNFα and IL-17 are inflammatory mediators that are elevated by periodontal infection, expressed by cells close to alveolar bone and stimulate RANKL expression and bone resorption^18^. A luciferase reporter assay demonstrated that TNFα and IL-17 induced NF-κB transcriptional activity in osteoblasts and osteocytes *in vitro* (Fig. 3a,b). TNFα stimulated NF-κB transcriptional activity through the canonical NF-κB pathway as shown by significant reduction of transcriptional activity when the p65 and p50 canonical NF-κB subunits were knocked down.

### Inhibiting NF-κB activation does not affect an inflammatory infiltrate induced by bacterial infection

**Figure 4:**
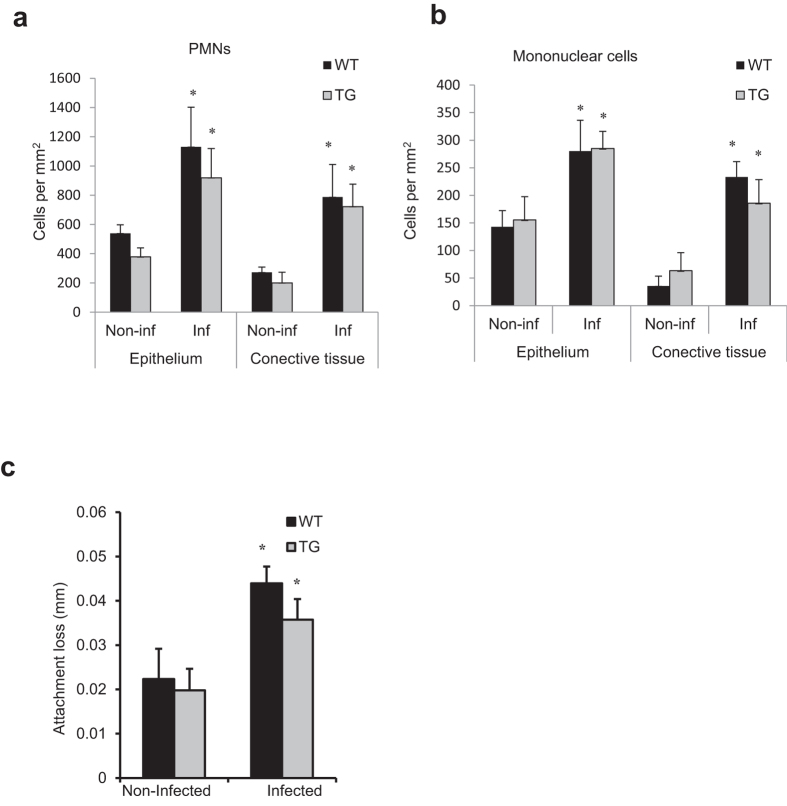
Inhibition of NF-κB in osteoblast lineage cells does not affect formation of an inflammatory infiltrate or loss of connective tissue attachment. PMNs and mononuclear cells in gingival epithelium or connective tissue were assessed in hematoxylin and eosin stained sections. Loss of connective tissue attachment to the molar teeth was measured in hematoxylin and eosin stained sections. ^*^Significantly different in infected compared to matched non-infected group (P < 0.05).

The effect of oral infection on formation of an inflammatory infiltrate was examined in WT and TG mice. Oral inoculation of bacteria stimulated a significant increase in the number of PMNs and mononuclear cells in the epithelium and connective tissue of the gingiva (P < 0.05) (Fig. 4a,b). However, there was no difference between WT and TG mice indicating that osteoblast lineage cells did not participate in recruitment of these leukocytes to gingiva (P < 0.05) (Fig. 4a,b). Infection stimulated a significant loss of connective tissue attachment, which is associated with bacteria-induced inflammation in the gingiva^19^. There was no difference in attachment loss between WT and TG mice (P > 0.05), consistent with a similar inflammatory infiltrate in both groups (Fig. 4c).

### Inhibition of NF-κB in osteoblast lineage cells decreases osteoclast numbers and reduces RANKL expression in osteoblast lineage cells

**Figure 5:**
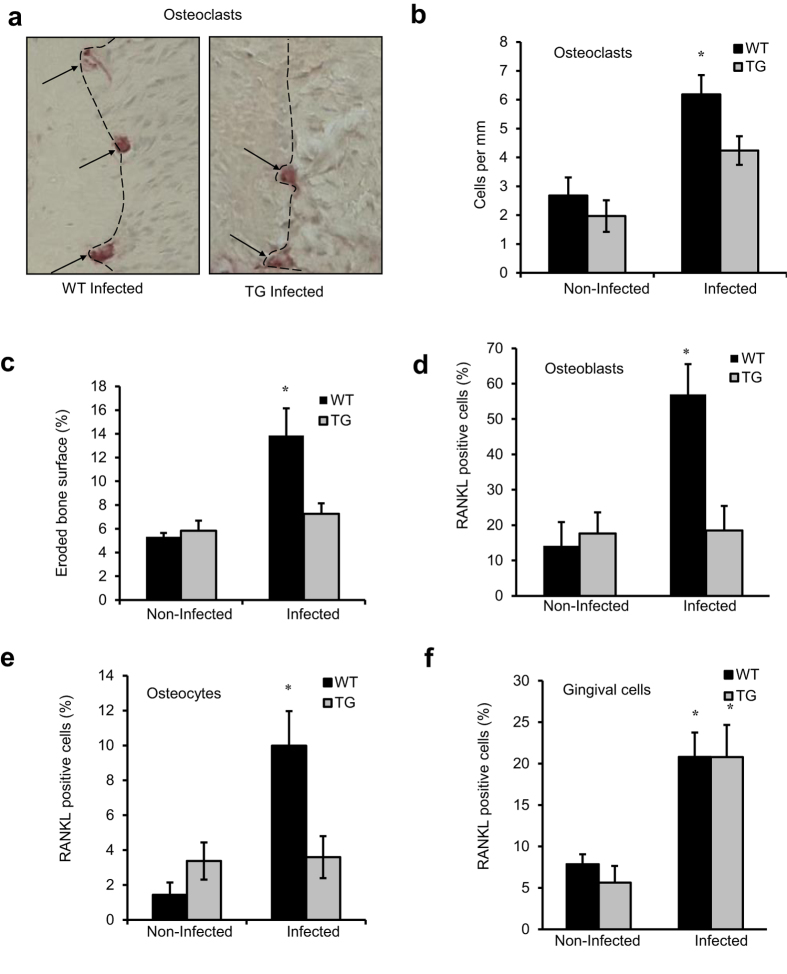
Inhibiting NF-κB activation in osteoblast lineage cells reduces osteoclast numbers and RANKL expression induced by periodontal infection. Periodontal disease was induced in wild-type and IKK-DN mice by oral inoculation of bacteria as described in Fig. 1. (**a**–**c**) Osteoclasts (indicated by arrows) were counted as multi-nucleated cells lining the bone surface in TRAP stained sections. Eroded bone surface was measured as the length of resorption lacunae divided by total bone length in TRAP stained sections. (**d**–**f**) The expression of RANKL by osteoblastic cells, osteocytes and cells in gingival connective tissue was determined by immunofluorescence following the approach described in Fig. 2. ^*^significantly different compared to matched non-infected group (P < 0.05).

Mice exposed to oral pathogens exhibited an increase in osteoclast formation (Fig. 5a,b). In WT mice the number of osteoclasts was induced approximately 2.5-fold. Infection stimulated a significant increase in osteoclasts in TG mice, but the amount was approximately one half that stimulated in WT mice (P < 0.05) (Fig. 5a,b). The percent eroded bone surface, reflecting osteoclast activity was reduced by 50% in TG compared to WT mice following infection (P < 0.05) (Fig. 5c). Osteoclast formation is largely driven by RANKL production, which is thought to be produced by leukocytes in the periodontium^20^. Unexpectedly we found that RANKL expression increased 4-fold in osteoblastic cells and 7-fold in osteocytes in WT mice after infection (P < 0.05). This increase was completely blocked in osteoblasts/osteocytes in TG mice (Fig. 5d,e). *In vitro* TNFα stimulated NF-κB nuclear localization in osteoblasts and osteocytes within 1 hour and RANKL expression within 24 hours (Supplemental Fig. S2 online). In contrast, RANKL production by cells in the gingiva was not affected by NF-κB inactivation in transgenic mice (P > 0.05) (Fig. 5f). Thus, periodontal infection stimulated RANKL production in osteoblast lineage cells in WT mice but not in TG, whereas other sources of RANKL were not affected in TG mice.

### Inhibiting NF-κB activation increases new bone formation

**Figure 6:**
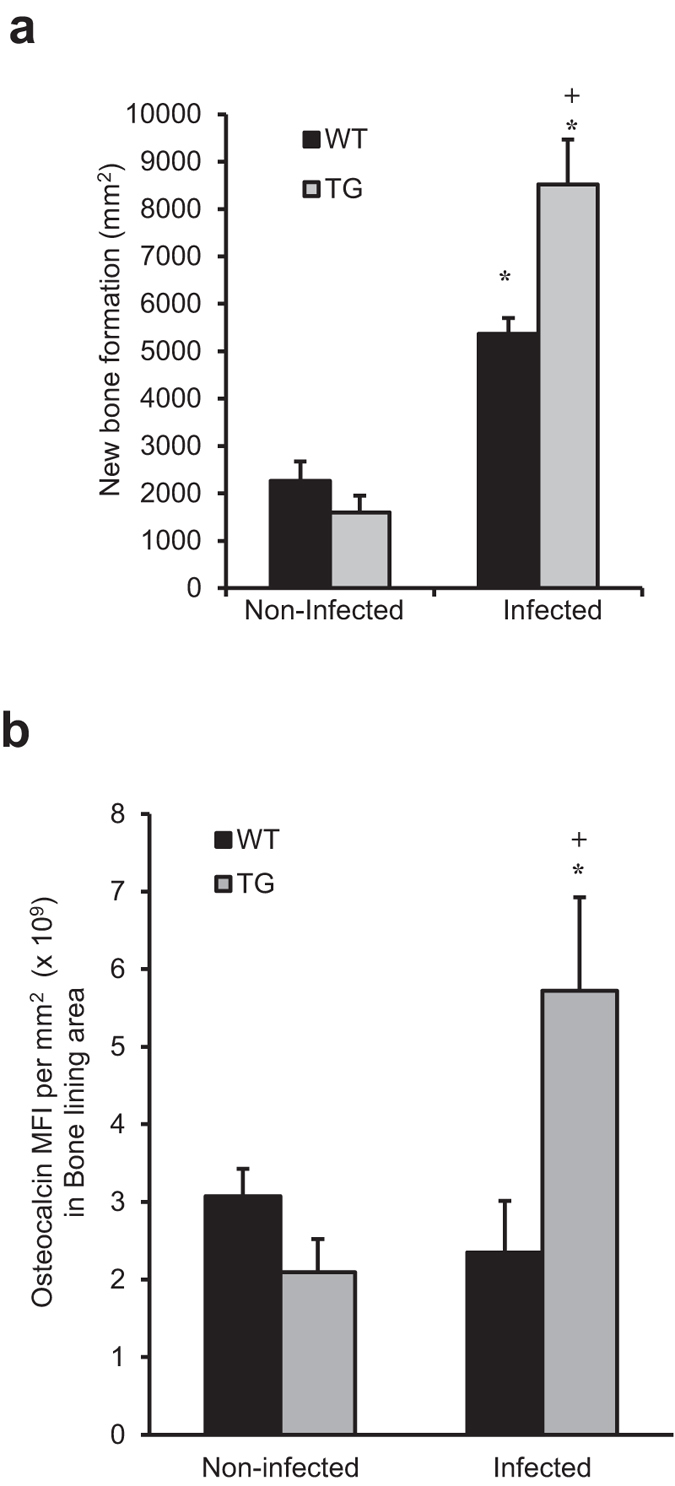
Inhibiting NF-κB in osteoblast lineage cells enhances new bone formation following induction of periodontal disease. Periodontal disease was induced in wild-type and IKK-DN TG mice by oral inoculation of bacteria as described in Fig. 1. (**a**) TRAP stained sections were analyzed for the amount of new bone formation using the reversal line as a guide. (**b**) Osteocalcin was measured by immunofluorescence with data expresses as the mean fluorescence intensity. ^+^significantly different in infected compared to matched non-infected group; ^*^significantly different in infected TG compared to infected WT (P < 0.05).

To determine whether there was a difference in the capacity of transgenic and WT mice to form new bone and repair the bacteria-induced bone loss, periodontal bone formation was measured. The amount of new bone formation (osteoid) reflecting osteoblast activity was measured histologically following oral infection and was 60% higher in the TG compared to the WT mice (P < 0.05) (Fig. 6a). It was also assessed by osteocalcin, a bone-specific matrix protein whose expression is indicative of new bone formation^21^. Osteocalcin was 2.4-fold higher in the TG compared to the WT mice after induction of periodontal disease (P < 0.05) (Fig. 6b, Supplemental Fig. S3 online). To better understand the impact of bone formation following infection the ratio of bone formed per osteoclast number was measured to assess the amount of bone coupling. In the transgenic mice the ratio of bone formation per osteoclast was 2-fold higher than the wild-type mice(P < 0.05). Thus, the amount of bone repair (bone coupling) following periodontal infection is significantly higher in the experimental TG mice which inhibition of NF-κB (Supplemental Fig. S4a,b online). Furthermore, it is important to take into account that periodontal bone density at the start of the experiments was similar in WT and TG mice (data not shown).

### Inhibiting NF-κB activation increases the number of osteoblastic cells

**Figure 7:**
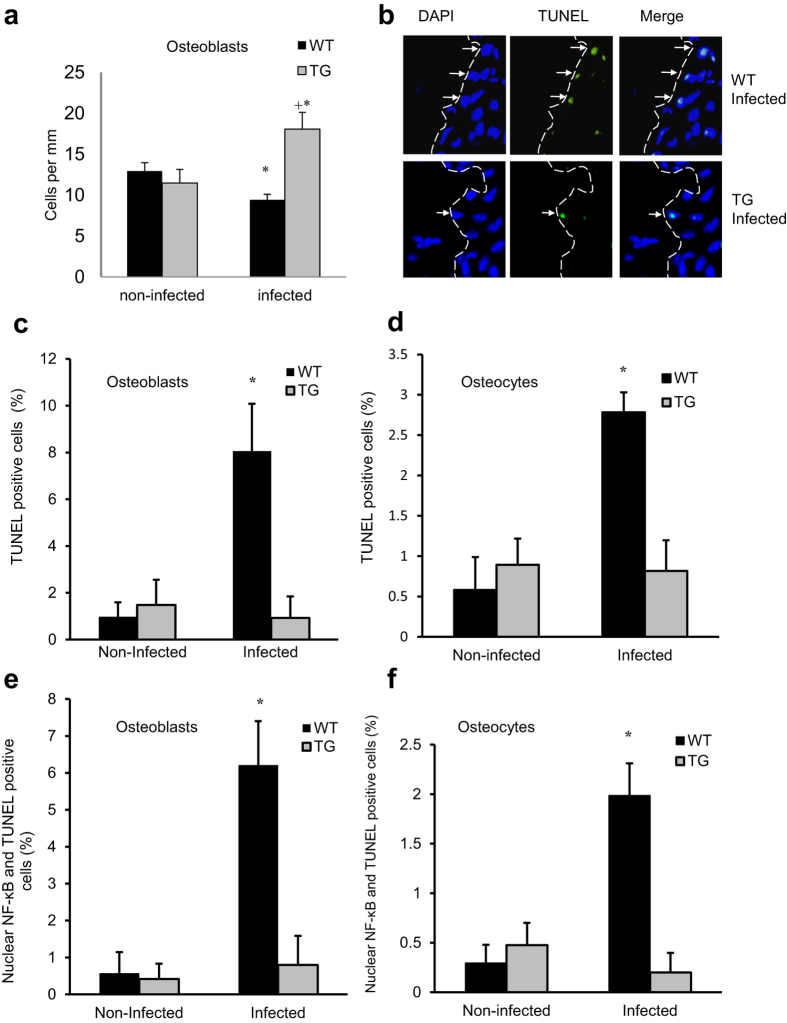
Periodontal infection reduces osteoblast numbers, which is reversed in transgenic mice with inhibition of NF-κB in osteoblast lineage cells. Periodontal disease was induced in wild-type and IKK-DN mice by oral inoculation of bacteria as described in Fig. 1. (**a**) The number of osteoblasts was measured per mm bone length by their characteristic cuboidal appearance in hematoxylin and eosin stained sections. (**b**–**d**) TUNEL^+^ osteoblasts and osteocytes were measured in in histologic sections with a fluorescent TUNEL assay. (**e**,**f**) The number of double positive (TUNEL^+^ nuclear NF-κB^+^) osteoblasts and osteocytes were measured by fluorescent TUNEL assay combined with immunofluorescence using an antibody to p65 following the approach as described in Fig. 2. Matched control antibody was negative. ^*^significantly different in infected compared to matched non-infected group (P < 0.05).

Oral infection decreased the number of osteoblastic cells by almost 30% in WT mice (P < 0.05) (Fig. 7a). However, the opposite occurred in TG mice with lineage specific inhibition of NF-κB. These mice experience a 57% increase in osteoblast numbers following infection (P < 0.05) (Fig. 7a). TUNEL assays were then carried out. Periodontal infection stimulated an 8.3-fold and 10.9-fold increase in the number of TUNEL positive osteoblasts and osteocytes cells, respectively in WT mice but no increase in TG mice (P < 0.05) (Fig. 7b–d). Furthermore, the link between NF-κB and apoptosis was further demonstrated by the 9.5-fold and 6.6-fold increase in osteoblastic and osteocytes cells that were double positive for TUNEL and for NF-κB nuclear localization in infected WT mice with no increase in the TG group (P < 0.05) (Fig. 7e,f). *O*steoblasts transfected with IKK had elevated annexin V and small dense nuclei consistent with apoptotic cells (Supplemental Fig. S5a,b online). This was unexpected since activation of NF-κB is typically associated with a reduction of apoptosis and an increase in survival^22^. However, the impact of infection on osteoblast apoptosis was consistent with the impact on osteoblast numbers in WT and TG.

## Discussion

We demonstrate for the first time that osteoblast lineage cells play an essential role in bacteria-induced periodontal bone loss. Blocking NF-κB activation in these cells prevented loss of bone induced by bacterial infection. The impact of periodontal inflammation on osteoblasts had two significant outcomes. Osteoblast lineage cells significantly contributed to bone resorption by increasing the number of osteoclasts and osteoclast activity. In addition, the capacity of osteoclasts to repair resorbed bone is significantly reduced by NF-κB. If NF-κB activation is suppressed in these cells there is no net bone loss even when periodontal infection stimulates an increase in osteoclasts. This is the first evidence that a critical component of periodontal bone loss is due to the effect of inflammation on bone formation and that osteoblast lineage cells play a role in periodontal bone resorption.

The expression of osteoclast inducing factors by leukocytes, particularly lymphocytes and monocytes is thought to be primarily responsible for inducing periodontitis^20^. RANKL in particular has been suggested to be the driving force for generation of osteoclasts in response to periodontal infection^23^. However, osteoblast lineage cells, also express RANKL and have an important role in stimulating osteoclastogenesis^17^,^23^. Osteocytes express RANKL to a greater degree than osteoblasts^24^. There is a direct relationship between RANKL and NF-κB as RANKL induces NF-κB through RANK signaling particularly in osteoclast precursors^25^. However, in many cell types inflammatory signaling activates NF-κB and induces RANKL, which is an important in inflammation-induced bone resorption^25^. Thus, we saw reduced RANKL expression in osteoblasts and osteocytes with dominant negative inhibition of NF-κB. In addition, MAP kinase and other signaling intermediates that are upstream of NF-κB may participate in stimulating RANKL expression^26^.

Although physiological bone resorption in bone remodeling is primarily regulated by cells of osteoblast lineage, particularly osteocytes, the contribution of these cells to inflammation induced bone loss is not well understood^17^. Infection stimulated increased NF-κB activation in osteoblasts and osteocytes of WT but not TG mice. However, similar basal activation of nuclear NF-κB was detected in both, which could be due to incomplete IKK inactivation by the dominant negative IKK, non-canonical NF-κB activation, or background immunofluorescence. We found that the number of osteoclasts was reduced approximately 50% when NF-κB activation is blocked in osteoclasts and that infection-induced osteoclast activity was significantly reduced. This agreed well with findings that infection stimulated RANKL production by osteoblasts/ osteocytes was blocked in the experimental mice but other sources were not affected, such as RANKL produced by cells in gingiva. Thus bone resorption still occurs in the dominant negative group but the resorbed bone is repaired by the activity of osteoblasts. As a result of resorption followed by repair there is no net change in the amount of bone in the DN group. In contrast the WT mice have more resorption and less reparative new bone formation and have net loss of bone. Another characteristic of periodontal disease is loss of connective tissue attachment of the gingiva to the root surface^1^,^2^. WT and TG mice had similar loss of connective tissue attachment, consistent with the concept that it does not involve osteoblast lineage cells. Thus, periodontal infection stimulates NF-κB activation in osteoblasts and osteocytes, and that these cell types are an important source of factors that stimulate bone resorption in periodontal disease.

Studies presented here demonstrate that an important feature of periodontal bone loss is the inhibition of bone formation. Infection caused a small decrease in osteoblast numbers in wild type mice. However, coupled bone formation was still greater in these mice, which may have been due to an increase in osteoblast activity, measured by histologic evidence of new bone matrix formed and expression of osteocalcin. Osteocalcin expression, which is incorporated into osteoid and reflects the level of bone formation, was significantly less in WT compared to TG mice. Thus, bone formation and osteocalcin production are promoted to repair loss of bone due to infection stimulated periodontitis^1^ and are greater when the response of osteoblast lineage cells to inflammation is reduced by dominant negative inhibition of NF-κB. These results are consistent with those obtained with ovariectomy induced osteoporosis^13^. In ovariectomy induced bone loss there was little change in osteoclast numbers caused by inhibiting NF-κB^22^. In contrast we found that in periodontal infection NF-κB inhibition reduced osteoclast numbers, consistent with changes in RANKL expression by osteoblast lineage cells. Infection also reduced osteoblast numbers and increased osteoblast apoptosis to a greater extent in WT than TG mice. This is consistent with findings that inflammation induced by periodontal pathogens *in vivo* causes apoptosis of osteoblasts^27^. Although NF-κB is typically anti-apoptotic, under conditions that enhance inflammation it has been shown to have an indirect pro-apoptotic effects through induction of apoptotic factors such as TNFα and Bcl-2-associated protein (BAX)^28^.

In summary, we report here demonstrating a critical role for osteoblasts in periodontal bone loss resulting from the effect of bacteria-induced inflammation on these cells mediated by NF-κB. We report for the first time that periodontal infection induces NF-κB in osteoblasts and osteocytes and that this activation is detrimental due to the impact of these cells on stimulating osteoclast formation and limiting reparative bone formation. Thus, these studies provide new insight into formation of osteolytic induced by inflammation, which is less well understood than physiologic bone remodeling. It may be possible to target NF-κB pathways to prevent inflammation induced bone loss by reducing bone resorption and enhancing bone formation.

## Material and Methods

### Animals

Transgenic male and female mice (Col1α1.IKK-DN) were generated that express a dominant negative IKK under the control of a 2.3 kbp element of the collagen 1α1 promoter that restricts activation of NF-κB in osteoblast lineage cells^12^,^14^,^15^. Mice received antibiotics (kanamycin and ampicillin to reduce the resident flora for 4 days and oral cavities swabbed for 2 days with 0.12% chlorhexidine gluconate rinse (Peridex, Procter and Gamble, Cincinnati, OH) prior to oral inoculation of bacteria. After 3 days without treatment and fasting for 3 hours, periodontitis was induced by inoculation of *P. gingivalis* and *F. nucleatum* once a day for 2 weeks around the molars and compared to mice inoculated with vehicle alone (2% methylcellulose). Mice were euthanized 6 weeks after oral inoculation. All animal procedures were approved by the Institutional Animal Care and Use Committee at the University of Pennsylvania. All methods were carried out in accordance with approved guidelines of the Institutional Animal Care and Use Committee.

### Preparation of histologic specimens

Specimens were fixed in 4% paraformaldehyde overnight at 4 °C and decalcified in 10% EDTA for 3–4 weeks. Paraffin-embedded histologic sections were prepared for the region between the 1^st^ and 2^nd^ and 2^nd^ and 3^rd^ molars that included the teeth, gingiva, bone and periodontal ligament as described^29^.

### Detection of polymorphonuclear cells (PMNs) and mononuclear cells in histologic sections

The number of PMNs and mononuclear cells in epithelium and gingival connective tissue adjacent to epithelium was determined in hematoxylin and eosin stained sections as described^30^.

### Bone measurements

Bone density was determined using a microcomputer tomography system (μCT35; SCANCO Medical, Bassersdorf, Switzerland). Bone percentage was measured as the area occupied by bone between two adjacent teeth. In hematoxylin and eosin (H&E) stained sections, distance to the bone crest was measured as the length form the cemento-enamel junction (CEJ) to the crest of the bone. Loss of connective tissue attachment was measured as described^19^. Osteoblasts were counted as cuboidal bone-lining cells in areas of bone remodeling^3^. Histologic sections were stained with tartrate resistant acid phosphatase (TRAP) as previously described^30^,^31^,^32^. Osteoclasts were identified as multinucleated, TRAP-positive bone-lining cells and eroded bone was determined as total lacunae length per total bone length^29^. New bone formation was measured using the reversal line as a reference as described^33^. Images from H&E and TRAP stained sections were captured with a Nikon Eclipse 90i microscope (Nikon, Melville, NY, USA) and NIS Elements-AR software (Nikon) was used for analysis.

### Detection of osteocalcin, NF-κB, TUNEL and RANKL

Paraffin sections were subjected to antigen retrieval in citrate buffer at 95 °C. Sections were incubated with antibody to osteocalcin (Takara, Mountain View, CA), NF-κB-p65 (Rockland, Gillbertsville, PA) or RANKL (Abcam, Cambridge, MA). Antibody was localized with biotinylated secondary antibody and avidin-biotin horseradish peroxidase complex (Vector Laboratories, Burlingame, CA). Antibodies were visualized using streptavidin Alexa-546 (Invitrogen, Carlsbad, CA) and counterstained with DAPI. Tyramide signal amplification (PerkinElmer, Waltham, MA) was used to enhance the signal. Apoptosis was detected using a TUNEL kit with fluorescent probe (Promega, Madison, WI). Fluorescent staining of cuboidal-shaped osteoblastic bone-lining cells, osteocytes in bone or cells in the gingival connective tissue was observed under 400× magnification. NF-κB nuclear localization was determined by co-localization of NF-κB-p65 immunofluorescence and DAPI nuclear staining on captured images using NIS-Elements software (Nikon). Cells that were immunopositive for cytoplasmic NF-κB-p65 were also determined by immunofluorescence in DAPI stained sections using NIS-Elements software with images. RANKL immunofluorescence was quantified in images captured at by measuring mean fluorescence intensity (MFI) of cells that had an osteoblastic appearance. Osteocalcin matrix was assessed by immunofluorescence in 0.02 mm of bone adjacent to the edge and the MFI measured. Immunofluorescent images were analyzed at 400× magnification.

### NF-κB transcriptional activity, nuclear localization and RANKL expression *in vitro*

MC3T3 osteoblasts and MLO-Y4 osteocytes were grown in MEMα media with 10% fetal bovine serum (FBS). Cells were transfected with a NF-κB-luc construct^34^ (Stratagene, La Jolla, CA) or empty vector alone. Some cells were also transfected with scrambled or siRNA (Santa Cruz Biotechnology, Santa Cruz, CA) to knockdown NF-κB subunits p65, p50 or RelB or NF-κB inhibitor BAY 11-7082 (Santa Cruz Biotechnology, Santa Cruz, CA). Cells were incubated 1 day with TNFα (10 ng/ml) or IL-17 (10 ng/ml) (Peprotech, Rocky Hill, NJ) for 24 hours. Luciferase activity was measured with a Dual-Luciferase Reporter Assay System (Promega, Madison, WI). NF-κB RANKL kinetic assay also begun after 12 h starvation followed by incubations with TNFα for 1, 8 and 24 h and compared with 24 h unstimulated cells. Immunofluorescence for NF-κB-p65 (Rockland, Gillbertsville, PA) or RANKL (Abcam, Cambridge, MA) was done, after 10% formalin fixation (Sigma-Aldrich, Saint Louis, MO), as described above. Cells were observed under 400× with a Nikon inverted fluorescent microscope (Melville, NY, USA) and NIS Elements-D software used for analysis. Nuclear translocation of NF-κB was counted; RANKL was measured by mean fluorescence intensity. The annexin V assay was carried out with MC3T3 cells transfected with IKK expression construct or pcDNA empty plasmid (Santa Cruz Biotechnology, Santa Cruz, CA) as described above to induce NF-κB activation. Annexin V was measured by Annexin V-Biotin Apoptosis Detection Kit (Abcam, Cambridge, MA) with DAPI nuclear staining.

### Statistical analysis

Statistical analyses were performed using SPSS software (SPSS, Chicago, IL). One-way ANOVA was used to compare the differences between groups at a given time point and to compare differences from baseline values with the other time points, associated with Tukey’s post-hoc test as well as the homogeneity of variance test. The significance level was set at P < 0.05. Experiments were analyzed by a double blind examiner with 6 to 7 specimens per group.

## Additional Information

**How to cite this article**: Pacios, S. *et al*. Osteoblast Lineage Cells Play an Essential Role in Periodontal Bone Loss Through Activation of Nuclear Factor-Kappa B. *Sci. Rep*. **5**, 16694; doi: 10.1038/srep16694 (2015).

## Supplementary Material

Supplementary Information

## References

[b1] Graves D. T., Li J. & Cochran D. L. Inflammation and uncoupling as mechanisms of periodontal bone loss. J Dent Res. 90, 143–53 (2011). 21135192 10.1177/0022034510385236 PMC3144100

[b2] Pihlstrom B. L., Michalowicz B. S. & Johnson N. W. Periodontal diseases. Lancet 366, 1809–20 (2005). 16298220 10.1016/S0140-6736(05)67728-8

[b3] Pacios S. . Bacterial infection increases periodontal bone loss in diabetic rats through enhanced apoptosis. Am J Pathol. 183, 1928–35 (2013). 24113454 10.1016/j.ajpath.2013.08.017 PMC5745547

[b4] Darveau R. P. Periodontitis: a polymicrobial disruption of host homeostasis. Nat Rev Microbiol. 8, 481–90 (2010). 20514045 10.1038/nrmicro2337

[b5] Yucel-Lindberg T. & Bage T. Inflammatory mediators in the pathogenesis of periodontitis. Expert Rev Mol Med. 15, e7 (2013). 23915822 10.1017/erm.2013.8

[b6] Hajishengallis G. Immunomicrobial pathogenesis of periodontitis: keystones, pathobionts, and host response. Trends Immunol. 35, 3–11 (2014). 24269668 10.1016/j.it.2013.09.001 PMC3947349

[b7] Boyce B. F. Advances in the regulation of osteoclasts and osteoclast functions. J Dent Res. 92, 860–7 (2013). 23906603 10.1177/0022034513500306 PMC3775372

[b8] Valerio M. S. . MKP-1 signaling events are required for early osteoclastogenesis in lineage defined progenitor populations by disrupting RANKL-induced NFATc1 nuclear translocation. Bone. 60, 16–25 (2014). 24269279 10.1016/j.bone.2013.11.012 PMC3945035

[b9] Yasuda H. . Osteoclast differentiation factor is a ligand for osteoprotegerin/ osteoclastogenesis-inhibitory factor and is identical to TRANCE/RANKL. Proc Natl Acad Sci USA 95, 3597–602 (1998). 9520411 10.1073/pnas.95.7.3597 PMC19881

[b10] Palumbo C., Palazzini S., Zaffe D. & Marotti G. Osteocyte differentiation in the tibia of newborn rabbit: an ultrastructural study of the formation of cytoplasmic processes. Acta Anatom. 137, 350–8 (1990). 10.1159/000146907 2368590

[b11] Bellido T. Osteocyte-driven bone remodeling. Calcif Tissue Int. 94, 25–34 (2014). 24002178 10.1007/s00223-013-9774-y PMC3947228

[b12] Abu-Amer Y. Nuclear factor-kappa B signaling and bone resorption. Osteoporos Int. 24, 2377–86 (2013). 23468073 10.1007/s00198-013-2313-x PMC3884829

[b13] Chang J. . Inhibition of osteoblastic bone formation by nuclear factor-kappa B. Nat Med. 15, 682–9 (2009). 19448637 10.1038/nm.1954 PMC2768554

[b14] Mina M. & Braut A. New insight into progenitor/stem cells in dental pulp using Col1a1-GFP transgenes. Cells Tissues Organs 176, 120–33 (2004). 14745241 10.1159/000075033

[b15] Braut A., Kollar E. J. & Mina M. Analysis of the odontogenic and osteogenic potentials of dental pulp *in vivo* using a Col1a1-2.3-GFP transgene. Int J Dev Biol. 47, 281–92 (2003). 12755333

[b16] Graves D. T., Kang J., Andriankaja O., Wada K. & Rossa C. Jr. Animal models to study host-bacteria interactions involved in periodontitis. Front Oral Biol. 15, 117–32 (2012). 22142960 10.1159/000329675 PMC3766715

[b17] O’Brien C. A., Nakashima T. & Takayanagi H. Osteocyte control of osteoclastogenesis. Bone 54, 258–63 (2013). 22939943 10.1016/j.bone.2012.08.121 PMC3538915

[b18] Da Costa T. A. . Inflammation biomarkers of advanced disease in nongingival tissues of chronic periodontitis patients. Mediators of Inflammation. 2015 (2015) 983782. doi: 10.1155/2015/983782. 26063981 PMC4439505

[b19] Bezerra B de B. . A.actinomycetemcomitans-induced periodontal disease promotes systemic and local responses in rat periodontium. J Clin Periodontol. 39, 333–41 (2012). 22313458 10.1111/j.1600-051X.2011.01847.x PMC3330439

[b20] Kawai T. . B and T lymphocytes are the primary sources of RANKL in the bone resorptive lesion of periodontal disease. Am J Pathol 169, 987–98 (2006). 16936272 10.2353/ajpath.2006.060180 PMC1698808

[b21] Li F., Wang X. & Niyibizi C. Bone marrow stromal cells contribute to bone formation following infusion into femoral cavities of a mouse model of osteogenesis imperfecta. Bone 47, 546–55 (2010). 20570757 10.1016/j.bone.2010.05.040 PMC2926210

[b22] Schaffler M. B., Cheung W. Y., Majeska R. & Kennedy O. Osteocytes: master orchestrators of bone. Calcif Tissue Int. 94, 5–24 (2013). 24042263 10.1007/s00223-013-9790-y PMC3947191

[b23] Chen B. . RANKL expression in periodontal disease: where does RANKL come from? Biomed Res Int. 2014 (2014), doi: 10.1155/2014/731039. PMC3955606 24719884

[b24] Nakashima T. . Evidence for osteocyte regulation of bone homeostasis through RANKL expression. Nat Med. 17, 1231–4 (2011). 21909105 10.1038/nm.2452

[b25] Yamashita T. . NF-kappaB p50 and p52 regulate receptor activator of NF-kappaB ligand (RANKL) and tumor necrosis factor-induced osteoclast precursor differentiation by activating c-Fos and NFATc1. J Biol Chem. 25, 18245–53 (2007). 17485464 10.1074/jbc.M610701200

[b26] Grabbe C., Husnjak K. & Dikic I. The spatial and temporal organization of ubiquitin networks. Nature reviews. Molecular cell biology. 12, 295–307 (2011). 10.1038/nrm3099 PMC3654194 21448225

[b27] Behl Y. . Activation of the acquired immune response reduces coupled bone formation in response to a periodontal pathogen. J Immunol. 181, 8711–8 (2008). 19050291 10.4049/jimmunol.181.12.8711 PMC2638096

[b28] Rai A. . Transcription factor NF-kB associates with microtubules and stimulates apoptosis in response to suppression of microtubule dynamics in MCF-7 cells. Biochemical Pharmacology. 93, 277–89 (2015). 25536174 10.1016/j.bcp.2014.12.007

[b29] Liu R. . Diabetes enhances periodontal bone loss through enhanced resorption and diminished bone formation. J Dent Res. 85, 510–4 (2006). 16723646 10.1177/154405910608500606 PMC2253683

[b30] Graves D. T., Oates T. & Garlet G. P. Review of osteoimmunology and the host response in endodontic and periodontal lesions. J Oral Microbiol. 3 (2011), doi: 10.3402/jom.v3i0.5304. PMC3087239 21547019

[b31] Pacios S. . Diabetes aggravates periodontitis by limiting repair through enhanced inflammation. FASEB 26, 1423–30 (2012). 10.1096/fj.11-196279 PMC3316902 22179526

[b32] Liu R. . Diabetes enhances periodontal bone loss through enhanced resorption and diminished bone formation. J Dent Res. 85, 510–4 (2006). 16723646 10.1177/154405910608500606 PMC2253683

[b33] Romano P. R., Caton J. G. & Puzas J. E. The reversal line may be a key modulator of osteoblast function: observations from an alveolar bone wound-healing model. J Periodontal Res. 32, 143–7 (1997). 9085225 10.1111/j.1600-0765.1997.tb01396.x

[b34] Ginzburg S. . Piperlongumine inhibits NF-κB activity and attenuates aggressive growth characteristics of prostate cancer cells. Prostate. 74, 177–86 (2014). 24151226 10.1002/pros.22739 PMC4052841

