# Supplementary material for: Osteoblast Lineage Cells Play an Essential Role in Periodontal Bone Loss Through Activation of Nuclear Factor-Kappa B
Source: Sci Rep. 2015 Dec 15;5:16694. doi: 10.1038/srep16694 (PMC4678879; doi:10.1038/srep16694)
Supplement: Supplementary Information [file srep16694-s1.pdf]

Osteoblast Play an Essential Role in Periodontal Bone Loss Through Activation of Nuclear Factor-Kappa B

Authors: Sandra Pacios[1*], Wenmei Xiao[1,2*], Marcelo Mattos[1*], Jason Lim[1], Rohinton S. Tarapore[1], Sarah Alsadun[1], Bo Yu [3], Cun-Yu Wang[3], and Dana T. Graves[1].

Affiliations:

[1] Department of Periodontics, School of Dental Medicine, University of Pennsylvania, Philadelphia, PA

[2] Department of Periodontology, School and Hospital of Stomatology, Peking University, Beijing, China

[3]Division of Oral Biology and Medicine, School of Dentistry, University of California, Los Angeles, CA

*First three authors contributed equally

Corresponding author:

Dana T. Graves, DDS, DMSc
Department of Periodontics
University of Pennsylvania
240 S 40th St, Levy 122
Philadelphia, PA 19104
E-mail: dtgraves@dental.upenn.edu

Key Words: bone loss, osteoblast, osteoclast, inflammation, infection, periodontitis, periodontium

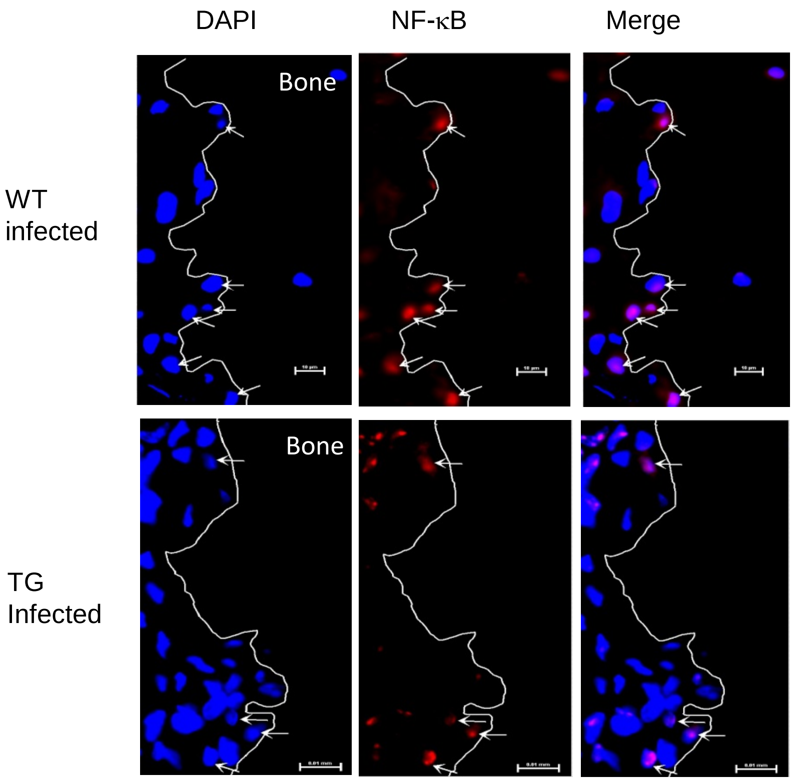

Immunofluorescence image on NF-κB. Periodontal disease was induced in wild-type and IKK-DN mice by oral inoculation of bacteria. Nuclear NF-κB in an WT and TG infected specimens, measured by immunofluorescence by co-localization of p65 (red), a subunit of NF-κB and DAPI (blue) nuclear staining. Arrows indicate osteocalcin positive osteoblastic cells lining bone with cuboidal nuclei that were not fusiform or parallel to the bone surface. Matched control antibody was negative.

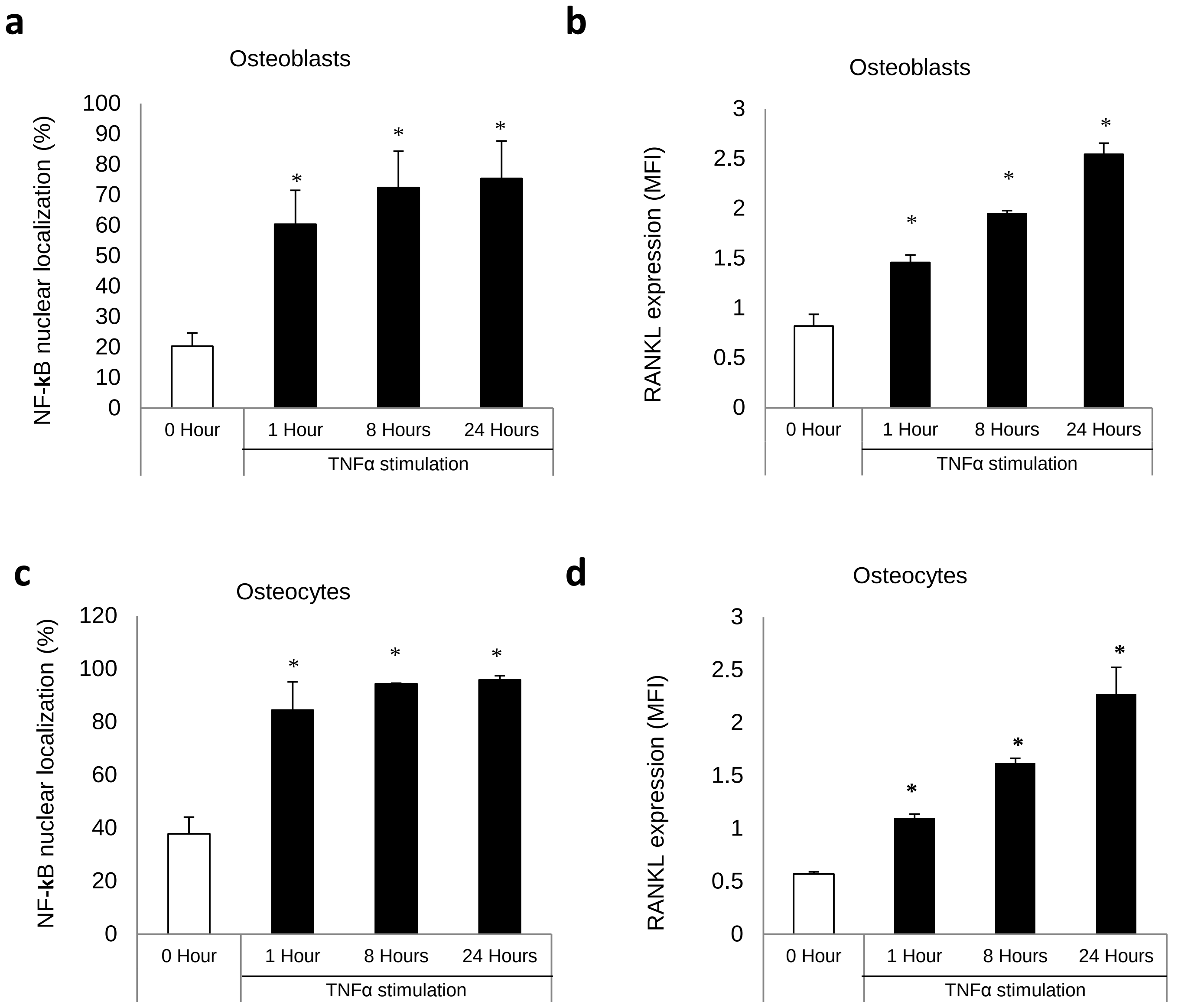

Kinetics of NF-κB and RANKL after TNFα stimulation. MC3T3 osteoblasts and MLO-Y4 osteocytes were incubated with TNFα as indicated. NF-κB nuclear localization was determined by immunofluorescence with antibody specific for p65 (red) and co-localization with DAPI (blue) nuclear stain. RANKL was measured by immunofluorescence and data collected as mean fluorescence intensity. Matched control antibody was negative. *, significantly different when compared to non-stimulated control.

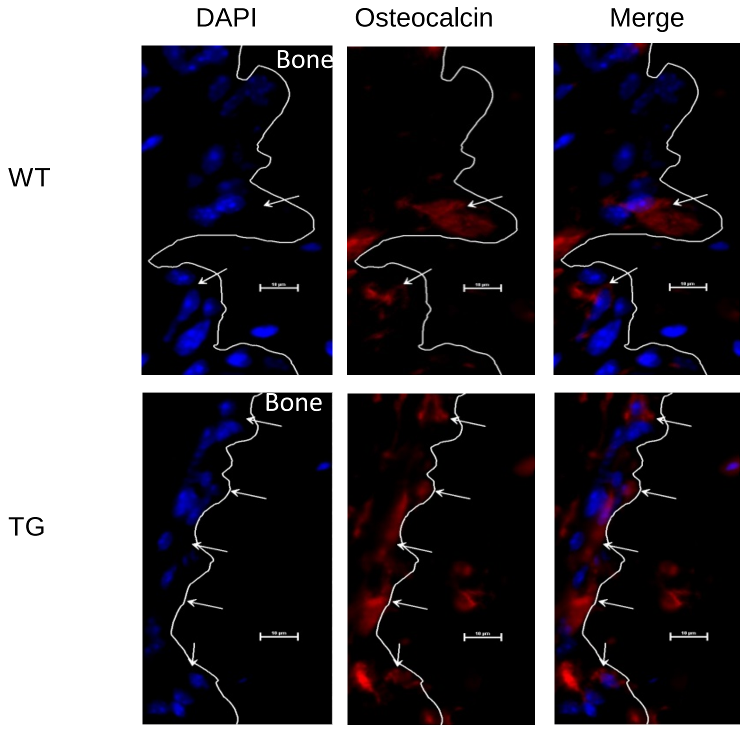

Osteocalcin expression. Osteocalcin was detected by immunofluorescence with specific antibody (red) in a WT and TG animals that had oral inoculation of periodontal pathogens. Sections were stained with DAPI (blue) nuclear stain. The white line indicates bone surface with periodontal ligament to the left. Arrows point to osteocalcin positive cells. White line indicates bone surface with periodontal ligament to the left and bone to the right. Matched control antibody was negative.

a

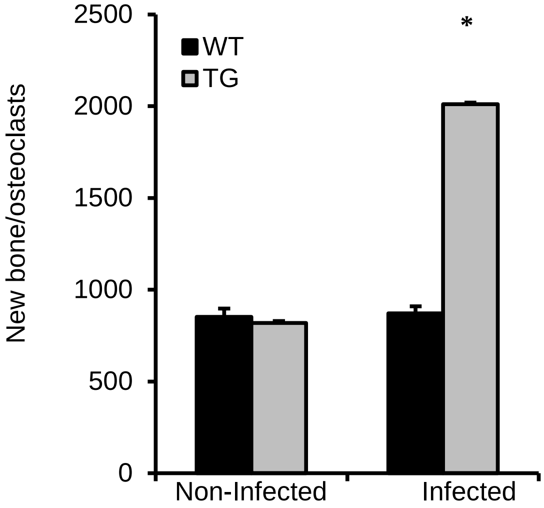

b

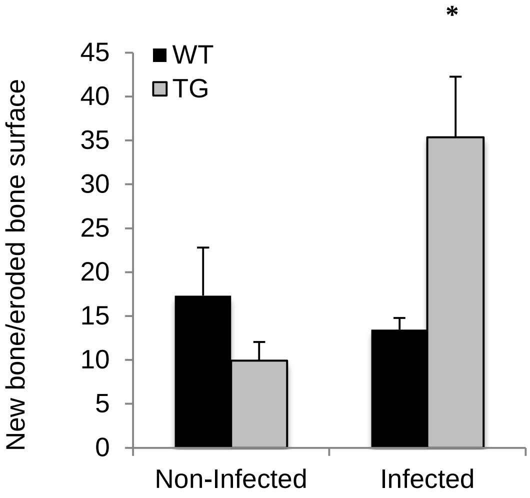

Assessment of bone formed in relationship to osteoclasts or osteoclast activity. Bone formed was assessed using the reversal line as a guide as described in Figure 6a and values were divided by the number of osteoclasts or eroded bone surface, measured in Fig 5. * significantly increased in infected TG mice (P<0.05).

a

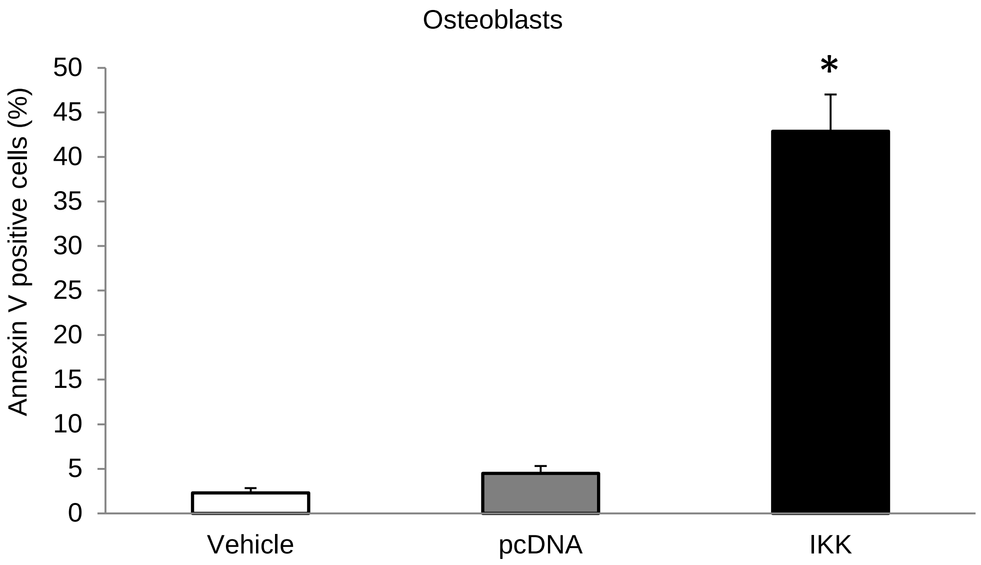

b

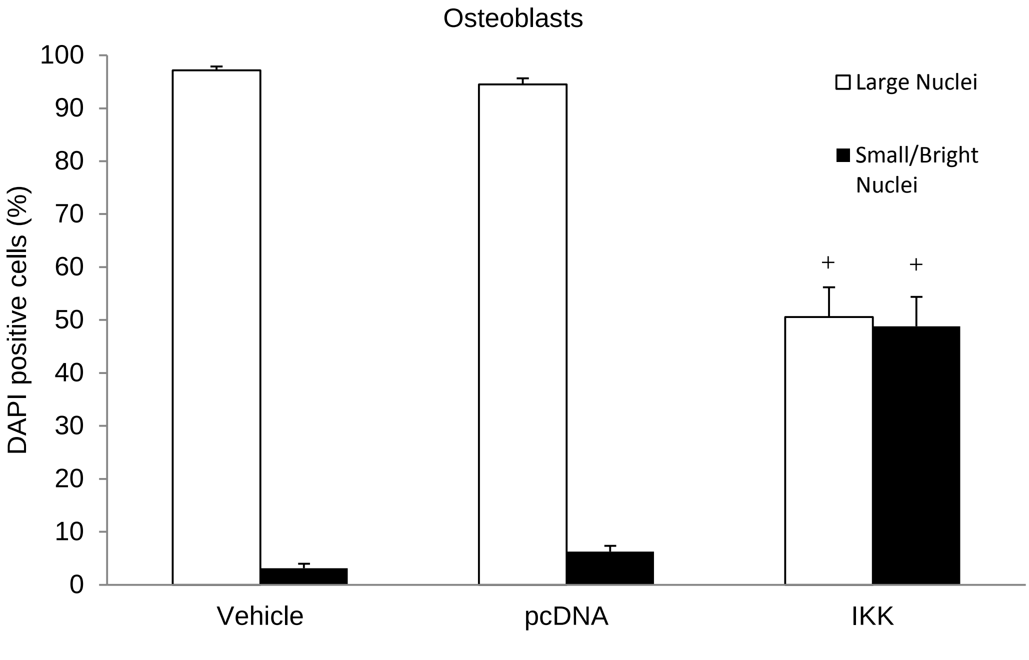

Annexin V expression and nuclei morphology in apoptotic MC3T3 cells. MC3T3 pre-osteoblastic cells transfected with IKK had significantly more annexin V+ cells than the control cells. It was also noticed that the cells that had brighter and smaller nuclei (based on the DAPI staining) were the cells that were undergoing apoptosis. * significantly different when compared to control.
